# An Investigation of the Immediate Effect of Static Stretching on the Morphology and Stiffness of Achilles Tendon in Dominant and Non-Dominant Legs

**DOI:** 10.1371/journal.pone.0154443

**Published:** 2016-04-27

**Authors:** Tsz-chun Roxy Chiu, Hiu-ching Ngo, Lai-wa Lau, King-wah Leung, Man-him Lo, Ho-fai Yu, Michael Ying

**Affiliations:** Department of Health Technology and Informatics, The Hong Kong Polytechnic University, Hung Hom, Kowloon, Hong Kong SAR, China; Universite de Nantes, FRANCE

## Abstract

**Aims:**

This study was undertaken to investigate the immediate effect of static stretching on normal Achilles tendon morphology and stiffness, and the different effect on dominant and non-dominant legs; and to evaluate inter-operator and intra-operator reliability of using shear-wave elastography in measuring Achilles tendon stiffness.

**Methods:**

20 healthy subjects (13 males, 7 females) were included in the study. Thickness, cross-sectional area and stiffness of Achilles tendons in both legs were measured before and after 5-min static stretching using grey-scale ultrasound and shear-wave elastography. Inter-operator and intra-operator reliability of tendon stiffness measurements of six operators were evaluated.

**Results:**

Result showed that there was no significant change in the thickness and cross-sectional area of Achilles tendon after static stretching in both dominant and non-dominant legs (p > 0.05). Tendon stiffness showed a significant increase in non-dominant leg (p < 0.05) but not in dominant leg (p > 0.05). The inter-operator reliability of shear-wave elastography measurements was 0.749 and the intra-operator reliability ranged from 0.751 to 0.941.

**Conclusion:**

Shear-wave elastography is a useful and non-invasive imaging tool to assess the immediate stiffness change of Achilles tendon in response to static stretching with high intra-operator and inter-operator reliability.

## Introduction

Achilles tendon (AT) is the largest and strongest tendon in humans. Its major function is to transmit the force produced by calf muscles to heel bone [1]. Although AT is primarily made up of the type-1 collagen fiber which has high strength and flexibility [2,3], exposure to excessive mechanical loadings during vigorous exercises including running and jumping may easily result in tendon injuries such as rupture and tendinopathy [3]. Ultrasound is a useful imaging tool for the assessment of musculoskeletal structures because of its high image resolution, non-invasiveness and real-time capability. Shear-wave elastography (SWE) is a new ultrasound technology which allows quantitative evaluation of soft tissue stiffness (Young’s Modulus) [4,5]. Its principle is to send acoustic radiation force impulses through soft tissues with a particular density (ρ) and to compute the tissue shear elasticity (μ) in kilopascal from the velocity of shear wave (cT) travelling within the tissue based on the equation: μ = ρcT^2^ [5]. SWE has been used for the assessment of breast and liver tissues [5]. However, there are only a few studies reported SWE assessment of AT, and they studied the characterization of normal and abnormal AT, repeatability of stiffness measurement, AT stiffness augmentation and stretched AT elastic anisotropy [4,5,6,7]. The feasibility of using SWE in the assessment of AT properties was supported by these studies due to good agreement in reported results [4] and fair repeatability in measurements [4,5,6,7] as well as clear morphological delineation [5].

Static stretching is commonly performed prior to athletic activities to avoid musculoskeletal injuries by reducing tendon stiffness and enhancing functional range of movement [8]. It has been reported that static stretching has no significant immediate effect on cross-sectional area (CSA) and slack length of AT [9,10]. However, the immediate effect of static stretching on AT stiffness is controversial in previous studies. Some studies reported a significant decrease in AT stiffness after static stretching [10,11,12], whilst Nakamura [13] found a significant increase in AT stiffness, and other studies showed no alteration in AT stiffness after static stretching [1,9,14]. The inconsistent results of previous studies may be attributed to the indirect and complex conventional methods of using grey-scale ultrasound and dynamometer for the measurement of AT stiffness in these studies.

With the advantages of real-time capability and quantitative measurement of soft tissue stiffness, SWE is an ideal imaging tool for accurate assessment of AT. However, the value of SWE in assessing the immediate effect of static stretching on AT has not been reported. Leg dominance arises from predominant loading of unilateral leg in specific activities [13]. Previous study has suggested the definition of leg dominance depending on the nature of activities–manipulative (kicking a soccer ball) or stabilization (standing on one leg) [14]. Tendons alter its structural and biochemical properties to adapt the magnitude and habit of mechanical loading during activities [15]. The asymmetric loading profiles between two legs may cause difference in tendon properties, such as higher AT stiffness in dominant leg [16]. Higher incidence of tendon rupture in left Achilles tendon was noted in which the left leg was considered as the dominant leg which supported body stability [1]. The mechanical properties of AT are different in dominant and non-dominant legs [1,15,16]. Static stretching is important for preventing and treating injuries, and is commonly used as a therapeutic tool in physical rehabilitation and sports [8]. Understanding the variation of the mechanical properties of AT between dominant and non-dominant legs in response to static stretching can help to devise appropriate treatment protocol for dominant and non-dominant legs. However, the effect of leg dominance on immediate effect of static stretching on AT properties has not been evaluated in previous studies.

With the use of SWE, the present prospective study aimed to investigate the immediate effect of static stretching on normal Achilles tendon (AT) properties; to compare the potential difference of the effect of static stretching on stiffness of AT between dominant and non-dominant legs, and to examine the inter- and intra-operator reliability of SWE measurements of AT stiffness. The primary aims of the study are to provide a new imaging perspective in determining immediate effect of static stretching on AT properties with the use of SWE, and support further research on the effectiveness of pre-exercise static stretching and designation of side-specific stretching programme to optimize athletes’ performance and prevent AT injuries.

## Methodology

### Subject Recruitment and Experimental Design

A total of 21 Chinese young healthy subjects were recruited on a voluntary basis via acquaintance. One subject had a previous history of lower leg injury and Achilles tendon rupture and thus the subject was excluded from the study. Finally, the remaining 20 subjects were included in the study (13 male and 7 female, mean age = 21.3 ± 1.4 years, mean height = 168.2 ± 8.0 cm, mean weight = 60.8 ± 9.6 kg, body mass index = 21.4 ± 2.5 kg/m^2^). All recruited subjects were informed on the study aims, examination procedures, rights of volunteers, and safety of ultrasound by an information sheet, and signed a written consent before the commencement of the study. The study was approved by the Human Subject Ethics Subcommittee of the Hong Kong Polytechnic University.

Achilles tendinopathy would affect the morphological and mechanical properties of AT [3], and may affect the effect of static stretching on AT stiffness [2]. Therefore, subjects who were symptomatic of Achilles tendinopathy and have dysfunction of Achilles tendon were identified. All subjects completed the Victorian Institute of Sports Assessment–Achilles questionnaire (VISA-A). The VISA-A evaluates the effect of tendinopathy on function and quantifies the symptoms and dysfunction of Achilles tendon [17]. According Robinson et al. [17], a recreational person with Achilles tendinopathy will have a VISA-A score of 70 or less. In addition, a patient with Achilles tendinopathy who has VISA-A score reached 70 indicated that he/she was cured. Thus, subjects with VISA-A score lower than 70 were excluded from the study. Other exclusion criteria were history of tendon rupture, trauma and surgery in lower leg, experiences of strength training or flexibility training [12], history of systemic, metabolic, endocrine and inflammatory diseases [18] and on hormonal treatment, corticosteroid drugs and contraceptive pills [1].

The leg dominance of subjects was determined by asking the subject the preferred leg to kick a ball [1]. It was suggested that the non-dominant leg refers to the preferred leg to kick a ball while the dominant leg is defined as the supporting leg for stability [1].

### Reliability Tests

To evaluate the inter-operator reliability of SWE measurement of AT stiffness, another 6 subjects were recruited and scanned by 6 operators. To evaluate the intra-operator reliability, each operator scanned the subjects three times in the same scanning session. The operator with the highest intra-rater reliability was selected to perform the ultrasound scanning in the main study, and performed the intra-operator reliability test of AT thickness and CSA measurements.

### Ultrasound examination of AT

In the main study, the 21 subjects were reminded to avoid vigorous exercises in lower limbs for at least 2 hours prior to the examination and were asked to rest for 30 minutes before pre-stretching ultrasound examination.

All ultrasound examinations were performed by the supersonic shear wave elastography ultrasound unit (Aixplorer; Supersonic Imagine, Aix-en-Provence, France) with a SuperSonic SuperLinear^™^ 4–15 MHz linear-array transducer. In the ultrasound examination of AT, the subject laid in a prone position on the examination couch with the feet hanging freely over the edge of the couch to avoid tendon stress. A customized ankle fixer was used to standardize the position of the ankle for ultrasound scanning. A generous amount of ultrasound gel was applied over the AT to form a gel gap which can ensure good probe-tissue coupling and prevent tissue distortion due to transducer compression. B-mode ultrasound and SWE were performed to assess the morphological and mechanical properties of AT respectively.

**Fig 1:**
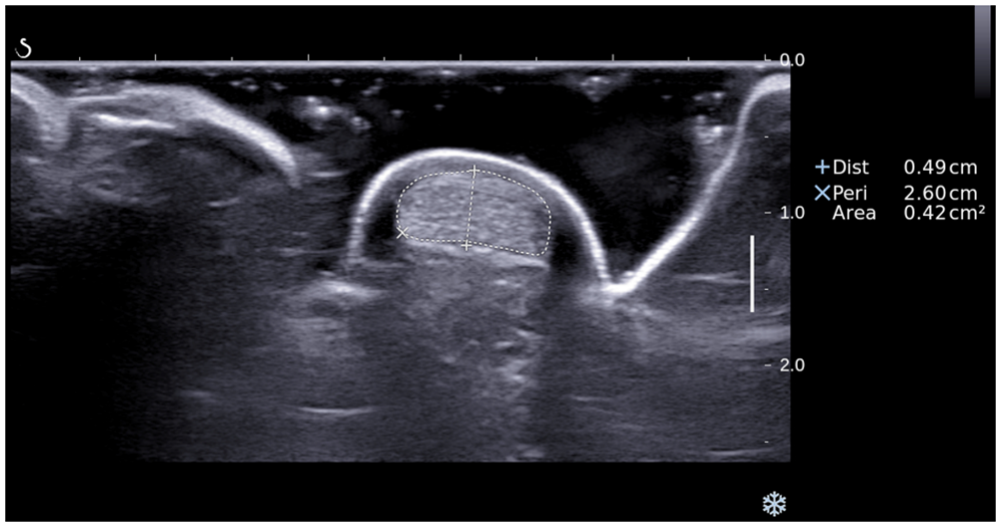
A transverse grey scale sonogram of Achilles tendon shows the measurement of the thickness (caliber +) and cross-sectional area (caliber ×) of the tendon.

In the B-mode ultrasound examination, the AT was scanned transversely until transverse scans at the level of medial malleolus were obtained. To ensure the ultrasound beam is perpendicular to the tendon with minimum anisotropy, the transducer was angled cranially and caudally until a scan plane which showed the maximum echogenicity of the tendon was obtained, and the thickness and cross-sectional area of the tendon were measured with the electronic calibers [1,5]. The tendon thickness was defined as the maximum anteroposterior diameter of the tendon, and the tendon CSA was measured by manual outline of the tendon boundaries (Fig 1) [1].

**Fig 2:**
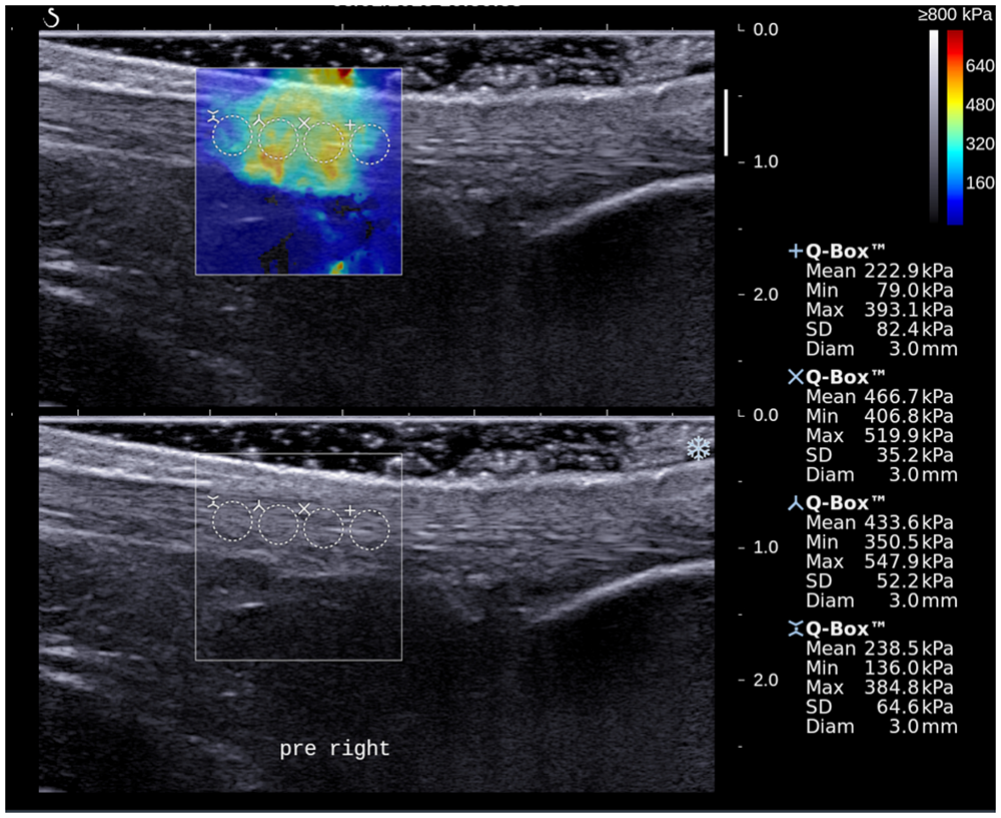
A longitudinal shear wave elastogram (upper) and the corresponding grey scale sonogram (lower) show an Achilles tendon on the non-dominant leg of a subject before stretching exercise. Four Q-boxes with a diameter of 3 mm were placed along the long axis of tendon for the measurement of tendon stiffness. Different stiffness parameters are shown on the right side of the image.

**Fig 3:**
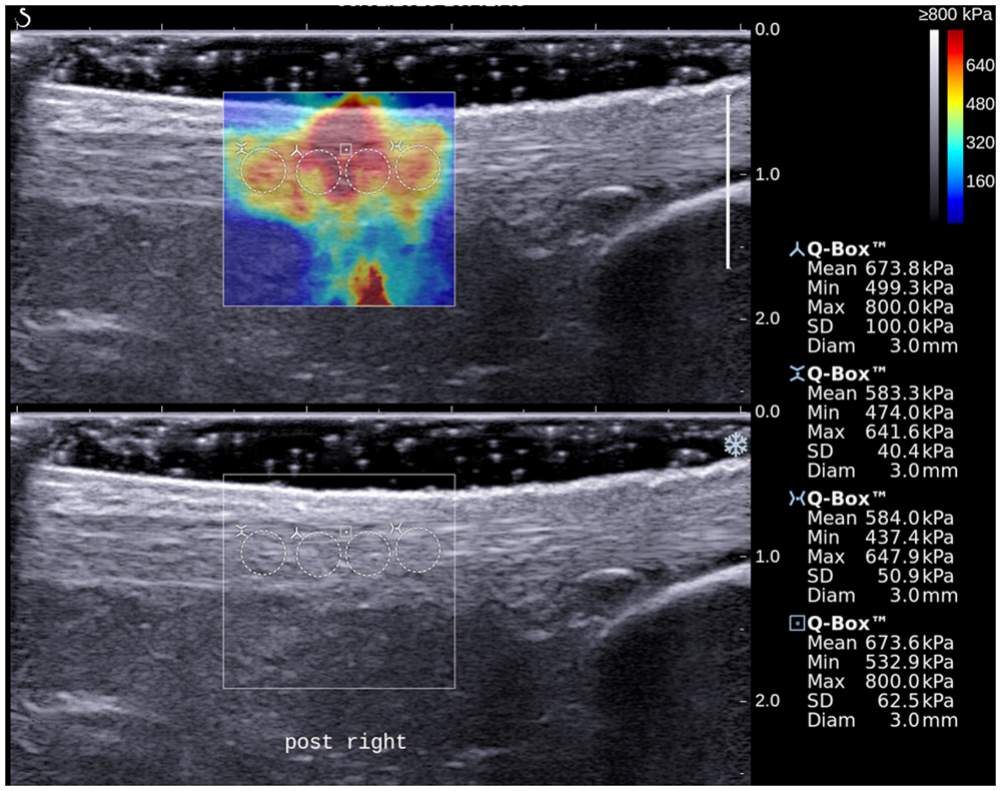
A longitudinal shear wave elastogram (upper) and the corresponding grey scale sonogram (lower) show the same Achilles tendon as in Fig 2 of the subject after stretching exercise. Similar measurement protocol as described in Fig 2 was used to measure the tendon stiffness. Note the stiffness parameters of the tendon were substantially higher after stretching.

With the SWE function of the ultrasound unit activated, the stiffness of AT of the subject was evaluated and the AT was examined with longitudinal scans. To standardize the region of AT to be assessed, the inferior border of the 1.56 cm x 1.56 cm acquisition box was placed 2 cm proximal to calcaneal insertion. SWE scanning was performed until the color display in the acquisition box became steady and homogenous, and the elastogram was obtained. For each AT, two shear-wave elastograms were obtained at different longitudinal planes of the tendon. On each elastogram, the measurement of tendon stiffness was performed using Q-box measurement tool which was an in-built program for automatic calculation of mean, maximum, minimum and standard deviation (SD) of stiffness values [5]. A total of 4 Q-boxes with a diameter of 3 mm were placed along the long axis of tendon in the image for the measurement (Figs 2 and 3).

### Stretching protocol

Each subject had a pre-stretching ultrasound examination on both legs. The subject then performed a 5-minute static stretching on the left leg. In the 5-minute static stretching, the subject stepped on a 30° inclined platform with the body trunk leaned forward until the lower leg was aligned vertically, and the subject maintained in this posture for 5 minutes [7]. Immediately after the stretching, a post-stretching ultrasound examination was performed on the left leg using the same scanning protocol. After the examination of the left leg, the same stretching and ultrasound protocols were performed on the right leg.

### Data Analysis

All statistical data were expressed as mean ± SD and the effect of static stretching was defined as the difference in tendon properties after stretching. For each AT, the thickness, CSA and mean stiffness were obtained and analyzed. The overall mean stiffness value of each AT was the average of the mean stiffness measured by the 8 Q-boxes in the two shear-wave elastograms.

Statistical Package of Social Sciences software version 20.0 (SPSS, version 20 for Windows, Chicago, IL) was used to calculate the results of all statistical tests with a 95% confidence interval. Intra-class correlation (ICC) was used to evaluate the reliability of AT properties measurements among and within the 6 operators. The normality of the data was analyzed by Shapiro-Wilk test. Data following or violating normal distribution are analyzed by paired t-test or Wilcoxon signed rank tests respectively. Paired t-test or Wilcoxon signed rank test was used to examine change in tendon properties after static stretching and to compare the difference in tendon properties between dominant and non-dominant legs.

## Results

In the 20 subjects included in the study, The VISA-A scores were above 70 (Range: 74–98). Seventeen subjects had the dominant leg on the left side whereas the other 3 subjects had a right dominant leg. The calculated statistical power for ultrasound stiffness measurement was 0.796 with the 20 subjects (G*power, version 3.1.5, Franz Faul, Uni Kiel, Germany).

The mean thickness and CSA of bilateral AT before and after stretching are summarized in Table 1 (S1 and S2 Files). There was no significant difference in the AT thickness and CSA before and after static stretching (p > 0.05).

**Table 1:** Effect of static stretching on Achilles tendon morphology and stiffness.

	Thickness (Dominant), cm	Thickness (Non-Dominant), cm	CSA (Dominant), *cm*^*2*^	CSA (Non-Dominant), *cm*^*2*^	Stiffness (Dominant), kPa	Stiffness (Non-Dominant), kPa
**Pre-Stretching**	0.565 ± 0.104	0.561 ± 0.078	0.464 ± 0.112	0.469 ± 0.089	*491.3 ± 86.9	*^#^392.6 ± 83.1
**Post-Stretching**	0.564 ± 0.103	0.564 ± 0.077	0.477 ± 0.104	0.467 ± 0.082	500.5 ± 92.8	^#^495.0 ± 96.1
**Percentage change (mean ± 1 SD)**	- 0.1 ± 3.8%	0.6 ± 3.8%	3.6 ± 9.3%	- 0.1 ± 4.5%	3.0 ± 15.4%	21.0 ± 13.5%

* p < 0.05 refers to differences between dominant and non-dominant legs

^#^ p < 0.05 refers to differences between pre- and post-stretching

The mean stiffness values of AT before and after stretching exercise are summarized in Table 1 (S3 File). The pre-stretching stiffness of dominant AT (491.3 ± 86.9 kPa) was significantly higher than that of non-dominant AT (392.6 ± 83.1 kPa) (p < 0.05) whilst such difference became insignificant after static stretching (p = 0.830). There was a significant increase in the stiffness value in the non-dominant legs (from 392.6 ± 83.1 kPa to 495.0 ± 96.1 kPa) after performing stretching exercise (p < 0.05). Although there was also increase in the stiffness of AT in the dominant legs but the difference was not significant (p > 0.05).

Results showed that the intra-operator reliability [ICC (3, k) score] of AT stiffness measurement ranged from 0.751 to 0.941, whereas the inter-operator reliability [ICC (2, k) score] among the 6 operators was 0.749. The intra-operator reliability of AT thickness and CSA measurements was 0.991 and 0.962 respectively.

## Discussion

In the present study, the CSA and thickness of AT did not show significant change immediately after static stretching. These findings were consistent with previous studies adopting the same stretching protocol with the present study [9,10]. Results indicated that static stretching does not have immediate effect on the morphology of AT.A significant difference in pre-stretched AT stiffness between dominant leg (i.e. 491.3±86.9 kPa) and non-dominant leg (i.e. 392.6±83.1 kPa) was found in the present study which is consistent with Bohm’s study [16], and their result showed significant higher resting stiffness in dominant leg than non-dominant leg (p<0.05). The inherent higher stiffness of dominant AT can be explained by unequal mechanical loading leading to higher shear stress caused by micro-tearing and promotion of gene expression for type-I collagen production. Different mechanical loading of dominant and non-dominant legs during daily activities may account for the different mechanical properties of AT between both legs [15,16,19]. Therefore, there was difference in AT stiffness before stretching between dominant and non-dominant legs.Achilles tendon consists of 30% collagen, 2% elastin, and 68% extracellular matrix [20]. Performing the function of stability and supporting, higher frequency of repeated mechanical loading on dominant AT had been suggested to contribute to more frequent intra-tendon micro-tearing, leading to subsequent increased blood supply and extracellular matrix content to the tendon for structural reconstruction, accounting for a higher hydrostatic pressure in the tendon region [19]. Stress is defined as function of force per unit area. Since no significant difference had been found for CSA of bilateral AT [18], increased hydrostatic force hence contributes to a higher stiffness of dominant AT. Increased loading at dominant AT had been suggested to stimulate expression of insulin-like growth factor-I production (IGF-I) which stimulates synthesis of type I collagen and cell proliferation, resulting in an increase in cross-linking along the tendon and therefore increased the stiffness of AT [21].

The AT stiffness in non-dominant legs was significantly increased after static stretching whereas there was no significant changes in AT stiffness in dominant legs after static stretching. The substantial stretching effect in non-dominant legs may be due to the significant difference in the pre-stretch stiffness of the tendon between dominant and non-dominant legs. It has been reported that strain magnitude applied to Achilles tendon must exceed a particular threshold in order to trigger adaptation effects on the mechanical properties of the tendon. The strains must be high enough to produce sufficient stimulus that beyond the strains triggered by the mechanical load applied during daily activities to trigger adaptation effects on the Achilles tendon [22]. Since the pre-stretch AT stiffness in dominant legs was higher than that in non-dominant legs, the strains produced by the 5-minute static stretching on the AT in dominant legs might not be higher than the mechanical load applied during daily activities and were not high enough to trigger adaptation effects on the mechanical properties of the tendon. However, the pre-stretch AT stiffness in non-dominant legs is lower. Therefore, lower magnitude of strain can produce sufficient stimulus to trigger adaptation effects on the mechanical properties of the Achilles tendon in non-dominant legs. The present study showed increased AT stiffness after static stretching, and this finding is in consistent with the results of previous studies. Using a machine for material tests to perform mechanical tensile test of rat’s Achilles tendon, de Almeida et al. [23] found that Achilles tendons with stretching were stiffer than those without stretching. Using dynamometer and grey scale ultrasound to measure the passive torque and the displacement of Achilles tendon after ankle dorsiflexion respectively, Nakamura et al. [13] found that there was a significant increase in Achilles tendon stiffness after static stretching.

Compared with the fair inter-operator reliability of SWE measurement of AT stiffness in Aubry’s study (inter-operator reliability is 0.46) [4], the inter-operator reliability of the AT stiffness measurement using SWE found in the present study (inter-operator reliability is 0.749) was higher than the previous studies. Compared with the highest ICC values in intra-operator reliability test for SWE measurement of AT stiffness in Peltz’s study (highest intra-operator reliability is 0.42) [7], the intra-operator reliability of the six operators in the present study ranged from 0.751 to 0.941, which is higher than Peltz et al. [7]. The improvement in intra-operator and inter-operator reliability in SWE measurement of AT stiffness in the present study may be due to the standardization of scanning protocol and the special equipment used to standardize the ankle position. Peltz et al. [7] suggested that the measurement repeatability is mainly influenced by the relative position of transducer and tendon. Angle between transducer and axis of tendon was suggested to be a factor influencing shear wave velocity in the tendon and leading to inaccurate calculation of Young’s modulus [4]. Ankle fixer and standardized measurement site (2 cm proximal to the AT insertion) were used for the measurement of AT stiffness in the present study which standardized the position of feet and degree of flexion of ankle throughout the scanning process. Therefore, higher measurement reliability was resulted. The significant improvement in the SWE measurement reliability in present study suggests that standardization of scanning protocol and technique is crucial for SWE measurement of AT stiffness so that reliable measurements can be obtained.

The present study demonstrated the asymmetry of stiffness change of dominant and non-dominant AT where non-dominant AT had significant increase in stiffness after static stretching. This finding is useful in the design of stretching protocol in which it should consider the difference in the AT stiffness between the dominant and non-dominant legs.

There are limitations in the present study. The sample size of the study was small and only young subjects were recruited. Different static stretching parameters including loading, dose, magnitude, torque, and number of stretching cycle and duration of stretching had been reported to have influence on tendon stiffness [1,2,9,10,11,12,13,14]. In the present study, only 5 minute continuous and 30 degree dorsiflexion stretching protocol was adopted. Further SWE investigations to assess the effect of the number of stretching cycles, stretching angles, magnitude and duration on AT stiffness are suggested. The present study investigated the immediate effect of static stretching but the delayed effect was not studied. Further studies to investigate the delayed effect of static stretching on AT are suggested.

In conclusion, SWE is a useful and reliable imaging tool for the assessment of immediate stiffness change of AT upon static stretching. Dominant leg AT has a higher baseline stiffness than non-dominant leg AT. A 5-minute static stretching with 30 degree dorsiflexion can lead to significant increase in non-dominant AT stiffness which may help enhance performance of manipulative activities. Asymmetry of stiffness change of bilateral AT provides scientific ground for supporting static stretching as a means to even out the inherent bilateral AT stiffness difference and provides insight for future studies to manipulate various stretching parameters in order to optimize athletes’ performance.

## Supporting Information

S1 File Minimal data set for AT thickness. (DOCX)

S2 File Minimal data set for AT cross-sectional area. (DOCX)

S3 File Minimal data set for AT stiffness. (DOCX)
