# Supplementary material for: An Investigation of the Immediate Effect of Static Stretching on the Morphology and Stiffness of Achilles Tendon in Dominant and Non-Dominant Legs
Source: PLoS One. 2016 Apr 27;11(4):e0154443. doi: 10.1371/journal.pone.0154443 (PMC4847758; doi:10.1371/journal.pone.0154443)
Supplement: S2 File — (DOCX) [file pone.0154443.s002.docx]

| Cross-Sectional Area (CSA) | | | | | | |  |  |
| --- | --- | --- | --- | --- | --- | --- | --- | --- |
|  | **Dominant Leg (cm²)** | | |  | **Non-Dominant Leg (cm²)** | | | |
| **Subject** | **Pre** | **Post** | **Change** | **% Change** | **Pre** | **Post** | **Change** | **% Change** |
| 01 | 0.44 | 0.44 | 0.00 | 0.0% | 0.41 | 0.46 | 0.05 | 12.2% |
| 02 | 0.37 | 0.42 | 0.05 | 13.5% | 0.41 | 0.39 | -0.02 | -4.9% |
| 03 | 0.41 | 0.41 | 0.00 | 0.0% | 0.45 | 0.44 | -0.01 | -2.2% |
| 04 | 0.42 | 0.55 | 0.13 | 31.0% | 0.42 | 0.43 | 0.01 | 2.4% |
| 05 | 0.42 | 0.42 | 0.00 | 0.0% | 0.41 | 0.41 | 0.00 | 0.0% |
| 06 | 0.34 | 0.41 | 0.07 | 20.6% | 0.40 | 0.40 | 0.00 | 0.0% |
| 07 | 0.58 | 0.57 | -0.01 | -1.7% | 0.63 | 0.62 | -0.01 | -1.6% |
| 08 | 0.52 | 0.54 | 0.02 | 3.8% | 0.47 | 0.42 | -0.05 | -10.6% |
| 09 | 0.41 | 0.42 | 0.01 | 2.4% | 0.40 | 0.40 | 0.00 | 0.0% |
| 10 | 0.49 | 0.47 | -0.02 | -4.1% | 0.47 | 0.46 | -0.01 | -2.1% |
