# Supplementary material for: An Investigation of the Immediate Effect of Static Stretching on the Morphology and Stiffness of Achilles Tendon in Dominant and Non-Dominant Legs
Source: PLoS One. 2016 Apr 27;11(4):e0154443. doi: 10.1371/journal.pone.0154443 (PMC4847758; doi:10.1371/journal.pone.0154443)
Supplement: S3 File — (DOCX) [file pone.0154443.s003.docx]

| Stiffness |  |  |  |  |  |  |  |  |
| --- | --- | --- | --- | --- | --- | --- | --- | --- |
|  | **Dominant Leg (kPa)** | | |  | **Non-Dominant Leg (kPa)** | | | |
| **Subject** | **Pre** | **Post** | **Change** | **% Change** | **Pre** | **Post** | **Change** | **% Change** |
| 01 | 459.0 | 508.4 | 49.40 | 10.8% | 487.7 | 648.7 | 161.00 | 33.0% |
| 02 | 601.5 | 375.7 | -225.80 | -37.5% | 521.2 | 608.3 | 87.10 | 16.7% |
| 03 | 408.6 | 330.8 | -77.80 | -19.0% | 327.4 | 486.2 | 158.80 | 48.5% |
| 04 | 570.6 | 607.0 | 36.40 | 6.4% | 454.8 | 571.7 | 116.90 | 25.7% |
| 05 | 506.1 | 526.2 | 20.10 | 4.0% | 385.2 | 418.3 | 33.10 | 8.6% |
| 06 | 516.6 | 543.5 | 26.90 | 5.2% | 365.8 | 603.2 | 237.40 | 64.9% |
| 07 | 346.1 | 405.9 | 59.80 | 17.3% | 360.6 | 434.8 | 74.20 | 20.6% |
| 08 | 420.2 | 508.8 | 88.60 | 21.1% | 426.4 | 427.1 | 0.70 | 0.2% |
| 09 | 539.2 | 574.3 | 35.10 | 6.5% | 276.7 | 452.9 | 176.20 | 63.7% |
| 10 | 555.4 | 632.1 | 76.70 | 13.8% | 398.8 | 595.9 | 197.10 | 49.4% |
